# Obesity-Related Cognitive Impairment: An Update Overview of Mechanisms and Treatments

**DOI:** 10.1017/erm.2026.10049

**Published:** 2026-05-04

**Authors:** Chuanyu Zhong, Kai Chen, Shu Lin, Yinqiong Huang

**Affiliations:** 1 Second Affiliated Hospital of Fujian Medical University, China; 2 Garvan Institute of Medical Research, Australia; 3 Endocrinology, Second Affiliated Hospital of Fujian Medical University, Quanzhou, China

**Keywords:** adipose tissue–brain axis, cognitive impairment, exosome, microbiome–gut–brain axis, neuroinflammation, obesity

## Abstract

**Background:**

Lifestyle changes and unhealthy eating habits have led to a sharp rise in obesity rates worldwide. Obesity is closely associated with a range of complications, including cognitive impairment and dementia. Accumulating evidence indicates that obesity negatively affects cognitive function and may increase the risk of neurodegenerative diseases. Conversely, cognitive dysfunction may further contribute to the development and progression of obesity. With growing attention in this field, obesity-related cognitive impairment has emerged as an important research focus at the intersection of metabolic and neurological disorders.

**Methods:**

This article reviews the potential mechanisms underlying obesity-related cognitive impairment and summarizes emerging therapeutic strategies.

**Results:**

The development and progression of obesity-related cognitive impairment involve multiple mechanisms, including insulin resistance, systemic and central inflammation, immune dysregulation, microcirculatory alterations and changes in neurotransmitters and synaptic plasticity. Recent studies have focused on the adipose tissue-brain axis and the microbiota–gut–brain axis, in particular, the targeted effects of extracellular vesicles released from adipose tissue and microbiota on the brain.

**Conclusions:**

This article systematically reviews the mechanisms underlying obesity-related cognitive impairment and presents novel therapeutic strategies.

## Introduction

Obesity is related to excessive accumulation of adipose tissue (Ref. 1). Obesity is a chronic, low-grade state of systemic inflammation characterized by increased adiposity, macrophage infiltration into adipose tissue and elevated levels of inflammatory mediators and cytokines. Additionally, obesity is closely linked to several adverse outcomes, including cognitive decline and a heightened risk of dementia (Ref. 2). The hippocampus and cerebral cortex may be particularly susceptible to inflammation in patients with obesity. The hippocampus, which plays a pivotal role in learning and memory, is believed to be a key mediator in the development of cognitive impairment (Ref. 3). Observational studies across diverse populations in Europe, North America, East Asia and Africa have demonstrated that obesity and its related metabolic disorders serve as significant risk factors for cognitive impairment and dementia (Ref. 4). A recent epidemiological and Mendelian randomization study identified an independent link between obesity and cognition, with visceral obesity and elevated body mass index (BMI) correlating with cognitive function in Asian populations (Ref. 5).

Cognitive impairment is a significant disorder or impairment of an individual’s cognitive abilities, manifested by difficulties or limitations in cognitive processes such as thinking, memory, learning, understanding and judgement (Ref. 6). Research indicates that obesity markedly enhances the likelihood of insulin resistance (IR) in the brain. Disruption of brain insulin signalling pathways and reduced glucose utilization lead to metabolic dysregulation, resulting in local neuronal damage and subsequent cognitive impairment (Ref. 7). In older adults, obesity exacerbates harmful microvascular responses, leading to impaired blood flow regulation, which subsequently impacts the delivery of oxygen and essential nutrients to the brain (Ref. 8).

In this comprehensive review, we explore the relationship between obesity and cognitive impairment, examining obesity as a risk factor for changes in the central nervous system (CNS) function and one of the mechanisms leading to cognitive impairment. We outline mechanisms including IR, systemic and neuroinflammation, immune system dynamics, microcirculatory alterations, changes in neurotransmitters and synaptic plasticity, the adipose tissue-brain axis, the microbiota–gut–brain axis, and the effects of extracellular vesicle (EV) from adipose tissue and microbiota on the brain. Their roles in the onset and development of obesity-related cognitive impairment are elucidated. Furthermore, we investigated strategies to mitigate obesity-related cognitive impairment by implementing effective lifestyle modifications, such as optimizing physical activity, dietary habits and sleep patterns, as well as pharmacological treatments, bariatric surgery and EV stem cell therapy, aiming to improve the health and well-being of patients with obesity-related cognitive impairment.

## Interaction between obesity and cognitive impairment

To clarify the connection between obesity and cognitive impairment, an 8-year observational cohort study followed more than 20,000 participants from the general community in the United Kingdom. These findings suggest an association between obesity and adverse cognitive function, supporting the role of obesity management in preventing late-life dementia and cognitive decline (Ref. 9). Furthermore, the relationship between BMI and cognitive function remains a subject of ongoing debate. A meta-analysis encompassing 29 studies, utilizing random effects and dose–response models, revealed that being underweight or obese during midlife, as well as underweight in later life, elevated the risk of cognitive impairment and dementia by factors of 1.39, 1.31 and 1.64 respectively. Conversely, being overweight or obese in later life was associated with a 21% and 25% reduction in risk respectively. Dose–response analysis was used to examine the relationship between BMI and cognitive function. The analysis indicated that the risk for overall cognitive dementia, AD and vascular dementia (VAD) significantly increased when midlife BMI exceeded 29, 30 and 32 kg/m^2^ respectively. In middle-aged and older populations, a late-life BMI below 27 kg/m^2^ was associated with a reduced risk of AD, while a BMI exceeding 39 kg/m^2^ led to an increased risk (Ref. 10). In addition, the relationship between abdominal obesity and cognitive impairment has become a recent research topic. The current studies mainly show that abdominal obesity increases the risk of cognitive impairment (Refs 11–14). A cross-sectional study involving 30,697 participants in a Taiwanese Biobank revealed a significant association between abdominal obesity as defined by waist-to-hip ratio (WHR) and poorer cognitive performance. Hip circumference and overall obesity (defined by BMI and percentage of body fat) were not significantly associated with cognitive performance (Ref. 15). Thus, obesity increases the risk of cognitive impairment to some extent, which is further amplified by abdominal obesity (Ref. 16).

Obesity is largely driven by excessive intake of saturated fats and sugars, which are fundamental components of the Western diet (WD). Evidence from studies suggests that WD, characterized by its high levels of saturated fats and sugars, is associated with pathological changes that heighten the selective vulnerability of the hippocampus and impair hippocampus-dependent learning and memory, and weaken behavioural control of hunger and satiety-related stimuli. These effects are hypothesized to promote further WD consumption, consequently resulting in obesity and potential cognitive decline, thereby creating a vicious cycle (Ref. 17). Recent research emphasizes the bidirectional relationship between obesity and cognitive impairments, highlighting how cognitive dysfunction can increase the risk of obesity. For instance, Parent et al. discuss how memory deficits can disrupt the processing of hunger and satiety cues, thereby promoting overeating and further weight gain (Ref. 18). Similarly, Yeomans et al. reinforce this perspective by demonstrating that habitual intake of a high-fat and sugar diet is linked to poorer memory and higher impulsivity, which complicates self-regulation of food intake (Ref. 19).

Moreover, it is essential to recognize that the effects of an HFD on cognitive function may occur independently of obesity itself. For instance, Attuquayefio et al. found that college students exposed to an HFD for just 4 days exhibited significant declines in performance on hippocampal-dependent cognitive tasks, despite not experiencing noticeable weight gain (Ref. 20). Similarly, Kanoski et al. reported that rodents fed an HFD displayed cognitive deficits independent of body weight increase. These findings imply that specific dietary components may exert direct adverse effects on cognitive function, suggesting that cognitive impairments can arise from dietary factors even before the onset of physiological changes typically associated with obesity (Ref. 21).

## Mechanisms of obesity leading to cognitive impairment

**Figure 1.**
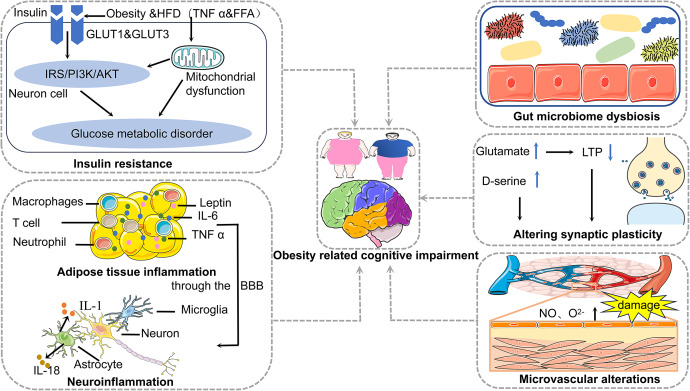
Mechanisms of obesity contributing to cognitive impairment encompass insulin resistance, adipose tissue inflammation, neuroinflammation, gut microbiome dysbiosis, synaptic plasticity alterations and microvascular alterations.

**Figure 2.**
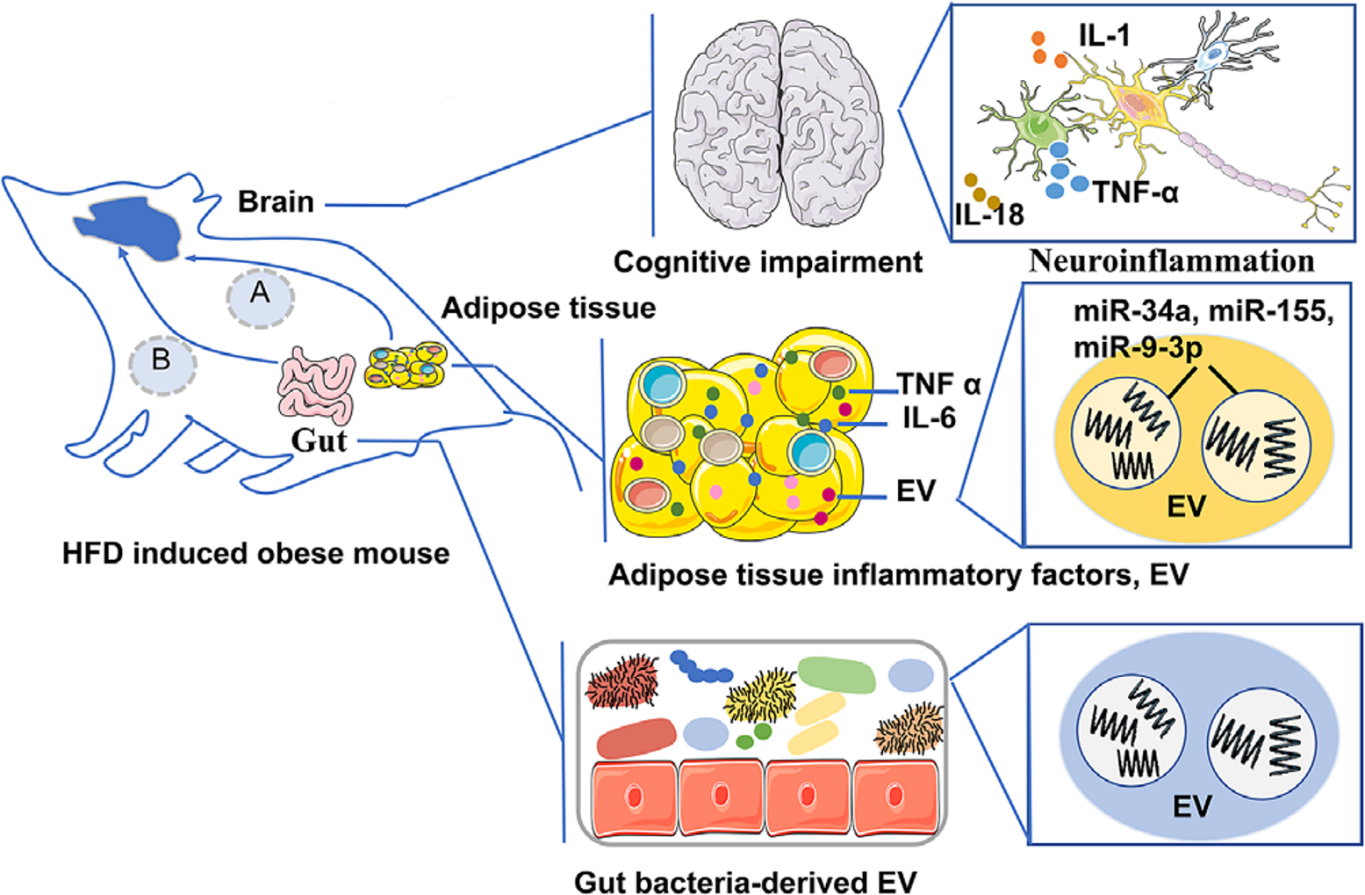
Pathway A is the adipose tissue-brain axis, which describes that in the obese state of mouse, the inflammatory factors derived from adipose tissue and EV cargo (miR-34a, miR-155, and miR-9-3p) enter the brain through the BBB, regulate neuroendocrine metabolism, lead to neuroinflammation and impair brain cognition; Pathway B is the microbiota–gut–brain axis, which describes that in the obese state of mouse the intestinal microbiota-derived EV and their cargoes that induce neuroinflammation and damage the BBB, promoting cognitive impairment.

Mechanisms of obesity contributing to cognitive impairment encompass insulin resistance, adipose tissue inflammation, neuroinflammation, gut microbiome dysbiosis, synaptic plasticity alterations and microvascular alterations (Figure 1). We also focus on the adipose tissue-brain axis and the microbiota–gut–brain axis, which are the mechanisms of obesity contributing to cognitive impairment (Figure 2).

### Insulin resistance

IR is the key link between obesity and cognitive impairment/AD (Ref. 7). Insulin exerts its cellular effects primarily through two major receptor pathways, the PI3-K and MAPK pathways. The PI3-K pathway is involved in regulating intracellular metabolic processes, whereas the MAPK pathway governs growth and cell division. Obesity-related insulin resistance is linked to disruptions in the PI3-K pathway, which plays a role in insulin’s metabolic functions (Ref. 22). Under normal conditions, activation of the insulin receptor leads to the phosphorylation of tyrosine residues on insulin receptor substrate-1 (IRS-1), initiating various downstream signalling cascades. However, in the context of obesity-induced metabolic disorders, increased levels of cytokines (e.g., TNF-α) and free fatty acids (FFA) can activate stress-activated kinases, resulting in the serine phosphorylation of insulin-like growth factor-1. This process contributes to the development of IR (Ref. 23).

Research has demonstrated that central IR is a key factor in cognitive decline (Ref. 24). Central insulin acts primarily on insulin-sensitive GLUT1 and GLUT3, rather than GLUT4 (Ref. 25). Insulin receptors are expressed in neurons and neuroglial cells, with a particular concentration in hippocampus(key cognitive region), as well as in the cerebral cortex, hypothalamus and olfactory bulb (Ref. 26). In healthy states, brain insulin plays a role in regulating mood, glucose metabolism, cognition, appetite and cerebral circulation (Ref. 27). In the early stages of IR, the resulting secondary hyperinsulinaemia leads to prolonged exposure of neurons to elevated insulin levels, potentially triggering neurodegeneration and irreversible memory deficits (Ref. 28). As a result, despite higher peripheral insulin levels, central insulin levels may decrease in the early phase of hyperinsulinaemia due to neuronal damage. This reduction is commonly associated with central IR and an increase in β-amyloid (Aβ) protein accumulation, which worsens central IR (Ref. 29). Furthermore, central IR facilitates the phosphorylation of tau proteins and facilitates the formation of neurofibrillary tangles. Additionally, Aβ can activate the mTOR signalling pathway, enhancing the synthesis of tau protein and mitochondrial activity. This cascading response ultimately leads to cognitive impairment, culminating in AD pathology (Ref. 30).

### Inflammation and immune system

Obesity is not only characterized as a metabolic disorder but also as an inflammatory condition that affects the function of the innate and acquired immune system. Typically, patients with obesity exhibit a mild state of systemic inflammation, which eventually spreads from peripheral tissues to the brain domain (Ref. 31). For instance, pro-inflammatory molecules released by adipocytes, such as leptin, IL-6 and TNF-α, are capable of crossing the blood–brain barrier (BBB) and inducing neuroinflammation. This process has been implicated in cognitive decline and the development of AD. In contrast, anti-inflammatory adipocyte factors, including adiponectin, IL-10 and apelin, are associated with lower levels of obesity and cognitive impairment (Ref. 32).

Neuroinflammation is one of the first key stress responses to occur within the brain and plays a significant role in the pathogenesis of AD. Microglia are important contributors to the neuroimmune defence and are the first cells to be activated and undergo proliferation when cerebral homeostasis is disturbed. (Ref. 33). The process of neuroinflammation involves microglial activation, with these cells able to polarize into either the pro-inflammatory M1 phenotype or the anti-inflammatory M2 phenotype, depending on the type of microenvironmental stress. M1 microglia release a variety of inflammatory cytokines and chemokines, which contribute to inflammation and neuronal damage. In contrast, M2 microglia are involved in tissue repair and maintenance, and they play a central role in the clearance of insoluble Aβ deposits. Numerous studies suggest that overactivation of microglia in response to Aβ and altered responses may have deleterious effects on neurons. Once over-activated, microglia exhibit characteristics of increased proliferation, initiate chemotactic responses and enhance the release of inflammatory M1 markers. Microglia release neurotoxic cytokines that can directly or indirectly damage neurons through activation of neurotoxic astrocytes (Refs 34, 35).

### Microcirculatory alterations

A dense network of microcirculation meets the higher metabolic demands of the brain. Cerebral microcirculation is responsible for the efficient delivery of oxygen, glucose and other nutrients to neural tissues, while also facilitating the removal of metabolic waste products. Additionally, it helps maintain ionic balance, supports the formation of the BBB and regulates the transport of various substances across the BBB (Ref. 36). Therefore, the condition of the microvasculature is vital for sustaining normal neuronal activity and cognitive function (Ref. 37). Evidences suggest that both obesity and aging contribute to structural and functional impairments of the cerebral microcirculation. Once obesity leads to cerebral microvascular dysfunction, pathological consequences, including endothelial dysfunction, neurovascular damage, microvascular rarefaction and BBB destruction, are induced (Refs 8, 38). Endothelial dysfunction associated with aging and obesity, characterized by decreased nitric oxide (NO) bioavailability, has been linked to cerebral hypoperfusion, which in turn contributes to cognitive impairment (Ref. 39).

Obesity induced by a WD is thought to selectively disrupt the BBB in the hippocampus, thereby impairing hippocampus-dependent learning and memory functions. This process has been corroborated by several studies. For instance, Hsu et al. highlighted a mechanistic link between WD intake and hippocampal damage, as well as dementia pathology resulting from BBB degradation (Ref. 40). Similarly, research by Hargrave et al. demonstrated that WD -induced BBB leakage in rats’ brains leads to alterations in spatial strategies, a change that may not affect BBB function in other brain regions. This suggests that while obesity may compromise hippocampal function, the effect might be localized, not uniformly damaging all brain regions (Ref. 41). Therefore, the WD and the ensuing obesity not only impair BBB integrity but also negatively affect the critical cognitive functions of the hippocampus.

### Neurotransmitters and synaptic plasticity

Synapses are connections between neurons that are important sites for information transfer. Synaptic plasticity, the capacity of neurons to modulate their connections, is closely linked to processes such as learning, memory and overall cognitive function. Long-term potentiation (LTP) and long-term depression (LTD) are the biological basis for the early stages of learning and memory. Alterations in synaptic plasticity, including changes in quantity, morphology and proteins, can lead to damage in specific brain regions, contributing to the development of certain neurodegenerative diseases (Ref. 42). An experiment inducing obesity in young and old C57BL/6 mice through chronic intake of a high-fat diet suggests that age-related obesity promotes hippocampal inflammatory responses, activates microglia, and may lead to synaptic dysfunction and cognitive impairment (Ref. 3). These studies suggest a bidirectional relationship between brain damage and synaptic plasticity.

An experimental animal study also has shown that long-term HFD-induced obesity significantly affects DNA methylation and gene expression in the hippocampus during ageing, particularly affecting genes related to neurodegeneration and metabolic processes (Ref. 43). Another study revealed that mice with obesity induced by an HFD exhibited differential expression of cyclic ribonucleic acid (circRNA) involved in endoplasmic reticulum stress and synaptic plasticity in hippocampal neurons. Notably, changes in circRNA occurred before histopathological alterations became apparent and could serve as an important reference, suggesting that it has the potential to be a biomarker of obesity-related cognitive impairment (Ref. 44).

Altered synthesis, storage, transport and degradation of neurotransmitters can lead to changes in synaptic plasticity and other neuronal impairment, ultimately resulting in cognitive impairment (Ref. 45). MCI and AD involve imbalances in glutamatergic activity, particularly N-methyl-D-aspartate(NMDA)receptor activity. Disruption of neurotransmission associated with NMDA receptors affects neuronal plasticity, which in turn affects cognitive and memory function. Among the modulators within the glutamatergic system, reduced levels of D-glutamate and elevated levels of D-proline lead to behavioural deficits, while elevated levels of D-serine are associated with cognitive decline. Notably, presynaptic glutamatergic synapses are enhanced in the early stages of MCI, but as the disease progresses, presynaptic glutamatergic neurons decrease dramatically (Ref. 46).

### Adipose tissue–brain axis

Adipose tissue acts as an endocrine organ and secretes a variety of signalling molecules involved in metabolic regulation, insulin signalling and inflammatory responses. Adipocytes produce leptin which acts upon hypothalamic neurons to maintain the balance between appetite and energy expenditure. Beyond metabolic effects, leptin also promotes neurogenesis and synaptic plasticity, linking it to processes of learning and memory, suggesting that leptin’s role may extend beyond systemic energy homeostasis (Ref. 47). Flores-Cordero et al. suggest that obesity can lead to leptin resistance, which subsequently impacts hippocampal function and may contribute to cognitive decline. Furthermore, reduced expression of leptin receptors in the cerebral cortex and hippocampus further increases the risk of AD (Ref. 48). Inflammation of adipose tissue is critical in the onset of obesity-related cognitive impairment, as it fosters an inflammatory microenvironment conducive to inflammasome complex formation. An experimental study showed that visceral adipose NLRP3 impaired memory functions through IL-1-mediated microglia activation. Visceral adipose NLRP3 is associated with obesity-induced cognitive impairment via IL-1R1 on CX3CR1^+^ cells. This suggests that the NLRP3/IL-1β signalling pathway could be fundamental to the connection between visceral obesity and cognitive decline in humans (Refs 49, 50). Beyond the traditional soluble factors, adipose tissue releases EV, which act as insoluble mediators of inter-organ communication, thereby modulating the function of the recipient organ (Ref. 51). EV consist of exosomes, microvesicles and apoptotic bodies that act as molecular mediators of intercellular communication. Exosomes are nano-sized membrane-bound vesicles that carry RNAs (mRNAs, miRNAs and other RNAs), proteins and lipids. Many of these exosomes play an important role in obesity and its complications (Ref. 52).

Pan et al. showed that miR-34a in adipocyte-released EV targets and represses the M2 polarization transcription factor Kruppel-like factor 4 (KLF4), leading to metabolic inflammation. Their study also showed that selective deletion of miR-34a in mice significantly reduced M1 macrophage populations and increased M2 macrophage populations within adipose tissue, accompanied by decreased levels of the M1 macrophage marker iNOS and increased levels of the M2 marker Arg1 (Ref. 53). Similarly, Zhang et al. found that miR-155 in microvesicles released from adipocytes modulates macrophage polarization, leading to M2 to M1 macrophages, triggering inflammation via Janus kinase (JAK)/signal transducer and activator of transcription (STAT) signalling (Ref. 54).

Furthermore, adipose tissue-EV may mediate brain function regulation. Brain aging often results in the decline of neuronal processes, such as sleep disorders and cognitive decline, which are major risk factors for various neurodegenerative diseases. To explore the mechanisms of cross-tissue regulation of brain aging by obesity, Li and his colleagues conducted experimental studies in which adipose tissue exosomes were extracted from fruit flies of different ages. They found that the expression of Circ_SXC in the exosomes was significantly downregulated with age. Their study showed that Circ_SXC could be taken up by the brain via the BBB, where it inhibited miR-87-3p through sponge adsorption, regulating the expression of neuroreceptor ligands (5-HT1B, GABA-B-R1, Rd1, Rh7, qvr and Na CP60E) and supporting normal synaptic signalling for brain function. However, as age increased, the downregulation of Circ_SXC in adipose exosomes disrupted this regulatory pathway, leading to age-related symptoms like sleep disorders. Mechanistic studies indicated that in the aged fruit fly brain, increased levels of dme-miR-87-3p and decreased expression of its target genes (5-HT1B, GABA-B-R1, Rdl, Rh7, qvr, Na CP60E) were associated with cognitive decline, memory impairment and SD (Ref. 55).

### Microbiome–gut–brain axis

The gut microbiome plays a pivotal role in the interplay between cognitive function and microbial composition (Ref. 56). Emerging evidence suggests that gut microbial diversity is intricately linked to cognitive health, with reduced microbial diversity often correlating with cognitive impairment. In this cross-sectional study, β-diversity, a measure of gut microbial community composition, was statistically significantly associated with all measures of cognitive function (Ref. 57). Moreover, a HFD has been shown to induce significant shifts in the gut microbiota, particularly by altering the firmicutes-to-bacteroidetes ratio, a hallmark microbial signature strongly implicated in obesity (Ref. 58). Obesity further exacerbates gut dysbiosis by promoting chronic low-grade inflammation and compromising intestinal barrier integrity, thereby disrupting host–microbiota homeostasis and adversely impacting metabolic regulation and cognitive function (Refs 59, 60). Beyond obesity, dietary composition, macronutrient profiles and host genetic predispositions independently influence the gut microbiome, with dietary factors emerging as principal determinants of microbial composition and function (Ref. 61). Collectively, gut microbiome alterations may arise as a consequence of obesity but are also dynamically regulated by dietary and environmental influences.

Numerous studies have indicated that the gut microbiota interacts closely with the gut itself, the enteric nervous system (ENS) and CNS. This microbiota communicates through pathways involving vagal signalling, the hypothalamic–pituitary–adrenal (HPA) axis and immune responses, all of which play critical roles in sustaining the microbiota–gut–brain axis (Refs 62, 63). The composition of the gut microbiota triggers the production of cytokines by the immune system. Macrophages and T cells respond to bacterial peptides from the gut by producing IL-1β and TNF-α. Furthermore, the microbiota supports specific CD4+ T cell subsets and stimulates immune pathways through antigenic interactions (Ref. 64). Short-chain fatty acids (SCFAs) activate and proliferate Foxp3 T regulatory (Treg) cells by modifying histones in the Foxp3 promoter region, while SCFAs enhance neuroinflammation by increasing retinoic acid production in the gut, reducing Th17 cell differentiation and promoting the growth of Tregs (Ref. 65). Conversely, long-chain fatty acids (LCFAs) promote Th1 and Th17 cell proliferation, along with TNF-α, IFN-γ and Csf2 production, especially in the context of multiple sclerosis. NLRP3 inflammasomes are activated in gut epithelium and bind to GPR43 and GPR109A by increasing the production of SCFAs in the intestinal microbiota. The NLRP3 inflammasome serves as an immunological link between gut microbial activity and brain function, facilitating brain modulation by the gut microbiota via NLRP3 signalling pathways (Ref. 62).

Notably, the gut microbiota also releases EV as a communication tool within the gastrointestinal environment (Ref. 66). These EV, secreted by gut bacteria, fungi and viruses, have compositions influenced by the microbiota profile, diet and host health status (Refs 67, 68). Gut microbiota-derived EVs have been shown to modulate host immune responses. For example, EV derived from the gut microbiota of mice induce differentiation of regulatory T cells and play a key role in maintaining immune homeostasis (Ref. 69). Studies have also shown that EV from *Akkermansia muciniphila* have a regulatory effect on metabolism and improve glucose tolerance in mice (Ref. 70). Additionally, there is growing evidence that EV from the microbiota of AD patients may induce tau hyperphosphorylation and aggregation in vitro, highlighting a possible pathway for disease progression. By inducing neuroinflammation and disrupting the BBB, these EV may contribute to cognitive decline in AD (Ref. 62). Therefore, future research may identify potential biomarkers for obesity-related cognitive impairment from gut microbiota-derived EV in obese patients.

## Treatment of obesity and cognitive function

Below are some treatments for improving obesity and cognitive function in humans (Table 1) and animals (Table 2), which include optimizing of lifestyle factors such as physical activity, diet and sleep, the use of anti-obesity drugs (GLP-1R agonists), anti-inflammatory drugs and consideration of bariatric surgery and EV stem cell therapy.

**Table 1.** Treatment for improving obesity or obesity related cognitive function in humans

Intervention/treatment	Target	Mechanisms of action	References
Aerobic physical activity	Metabolism nervous system	Promoting angiogenesis, enhancing cerebral vascular capacity, increasing cerebral blood flow, improving the delivery of oxygen and glucose, regulating inflammatory responses. Elevating cognitive reserve, fostering neurogenesis and synaptogenesis, enhancing structural integrity, volume, plasticity and connectivity within the brain.	(Refs 71–73)
Mediterranean diet	Metabolism nervous system	May prevent telomere shortening, reducing the risk of cognitive impairment, and exerting positive influences on both the morphology and functionality of the brain.	(Refs 74, 75)
Sufficient sleep	Metabolism nervous system	Impacting insulin resistance through central autonomic pathways, endocrine responses (such as changes in ghrelin and leptin) and inflammatory status. Altering the gut microbiota.	(Refs 76, 77)
Anti-obesity drug: GLP–1R agonists	Metabolism nervous system	Losing weight, protecting the neurons and reducing amyloid plaques.	(Refs 78, 79)
Anti-inflammatory drug (NSAIDs): diclofenac and fenamate	Nervous system	Inhibiting the release of pro-inflammatory mediators from microglial cells. Downregulating IL–1β, TNFα and transcription factor specificity protein 1.	(Ref. 80)
Bariatric surgery	Metabolism	Restoration of the leptin JAK/STAT signalling pathway.	(Ref. 81)

**Table 2.** Treatment currently applied in animal experiments to improve obesity or obesity-related cognitive function

Intervention/treatment	Target	Mechanisms of action	References
Anti-inflammatory drug: gold nanoparticles (AuNPs)	Nervous system	Alleviating neuroinflammation by inducing macrophage polarization towards the M2 phenotype, reducing the expression of pro-inflammatory cytokines, blocking leucocyte adhesion and reducing oxidative stress.	(Ref. 82)
Extracellular vesicles derived from NSPCs (exo-NSPC)	Nervous system	Rescuing the activation of IRS–1/FoxO and counteracting the reduction in stem cell proliferation and aging.	(Ref. 83)
EVs with anti-Trim21 oligonucleotides	Metabolism	UBE2M deficiency inhibited the neddylation of E3 ubiquitin ligase TRIM21 at positions K129/134, leading to reduced recruitment and ubiquitination-mediated degradation of E3 ubiquitin ligase VHL. Subsequently, VHL reduced HIF–1α-induced IL–1β production by degrading HIF–1α.	(Ref. 84)
Adipose-derived stem cells (ADSCs)	Nervous system	Regulating M2 polarization of microglia through the promotion of the PI3K/AKT signalling pathway.	(Ref. 85)
Probiotic mixture	Metabolism Nervous system	Altering the composition of the gut microbiota, augmenting the expression of genes associated with inflammation and neural plasticity in brain tissue.	(Ref. 86)
Bifidobacterium	Metabolism Nervous system	Promoting cognitive processes.	(Ref. 87)
*Akkermansia muciniphila*	Metabolism Nervous system	Anti-obesity, anti-inflammatory, anti-ageing and anti-neurodegenerative effects.	(Ref. 88)
Dimethyl itaconate	Metabolism Nervous system	Exhibiting cognitive enhancement in mice subjected to an HFD induction.	(Ref. 89)
Dietary restriction of leucine	Metabolism Nervous system	Improving obesity-related cognitive deficits through modulation of the microbiota-gut-brain axis.	(Ref. 90)
Butyrate	Metabolism Nervous system	Butyrate exerts its effects by ameliorating cognitive decline in a quinolinic acid-induced obesity model.	(Ref. 91)
FSH inhibitor	Nervous system	Suppressing the neuronal C/EBPβ-δ secretion pathway, that alleviated the AD-like phenotype in these mice.	(Ref. 92)
The deletion of the Ffar3 gene in Tg2576 mice	Metabolism Nervous system	The absence of FFAR3 reinstates the heightened brain metabolism observed in Tg2576 mice, suggesting that FFAR3 may serve as a potential novel target.	(Ref. 93)

### Behavioural intervention

A cross-sectional study suggests that engaging in aerobic exercise is linked to improved overall cognitive function and frontal lobe function in older adults who are overweight or obese. Physical activity can be used as an effective intervention to help lower the likelihood of cognitive decline or dementia in this demographic (Ref. 71). Physical activity and exercise not only promote angiogenesis, enhance cerebral vascular capacity, increase cerebral blood flow and improve oxygen and glucose delivery, but also modulate inflammatory responses. Simultaneously, they help to improve cognitive reserve, including promoting neurogenesis and synapse formation, enhancing structural integrity, volume, plasticity and connectivity within the brain. Together, these positive effects produce significant benefits to an individual’s cognitive function (Refs 72, 73).

Diet is an indispensable component, and research has shown that stronger adherence to the Mediterranean diet, compared to the WD, can significantly decrease the risk of developing all-cause dementia, AD or MCI. Mechanistic research suggests that the Mediterranean diet may prevent telomere shortening, a process associated with a reduced risk of various age-related diseases, including cognitive decline. In addition, adherence to the Mediterranean diet is inversely related to various AD biomarkers, positively affecting both brain morphology and function (Refs 74, 75). Furthermore, sleep plays a crucial role in human physiology, and sleep deprivation may affect insulin resistance through autonomic central pathways, hormonal changes (e.g., changes in gastrin and leptin), and inflammatory states. Evidence from rodent models indicates that sleep deprivation may alter the gut microbiota, which could contribute to the development of IR (Ref. 76). A 4-week fragmented sleep study in mice found reversible changes in the gut microbiota with increased food intake (Ref. 77). Thus, maintaining physical activity, a Mediterranean diet and good sleep is beneficial in improving obesity-related cognitive impairment.

### Clinical drug treatment

#### Anti-obesity drugs

Studies have shown that liraglutide, semaglutide and tirzepatide (a GLP-1/GIP dual agonist) demonstrate positive effects in weight management (Ref. 94). Additionally, tirzepatide has been shown to improve cognitive function through their neuroprotective properties (Ref. 78). A systematic review and meta-analysis by Kong et al. revealed that GLP-1 receptor agonists significantly improve outcomes in experimental AD models, including reducing amyloid plaques and enhancing learning and memory (Ref. 79).

Despite the notable therapeutic benefits of these drugs, potential adverse effects should also be considered, such as gastrointestinal reactions, the risk of hypoglycaemia and the potential for pancreatitis (Refs 95, 96). Therefore, it is crucial to balance the benefits and risks in clinical applications to optimize treatment strategies.

#### Anti-inflammatory drugs

In recent years, there has been growing interest in the protective effects of non-steroidal anti-inflammatory drugs (NSAIDs), such as diclofenac and finasteride. In a large retrospective cohort study, diclofenac significantly reduced the incidence of AD compared with other NSAIDs (aspirin, celecoxib, ibuprofen and naproxen). Diclofenac and fenamate-class drugs have similar chemical structures, and research from cell and animal models suggests that fenamate-class drugs participate in multiple pathways, including downregulating IL-1β, TNFα and transcription factor specificity protein 1. They also inhibit pro-inflammatory mediator release from microglial cells, thereby reducing the pathological changes in AD (Ref. 80).

Gold nanoparticles (AuNPs) are an emerging class of nanomaterials with excellent physicochemical properties, antioxidant and anti-inflammatory effects. These nanoparticles may alleviate neuroinflammation by inducing macrophages from a steady phenotype to the M2 phenotype, lowering the expression of pro-inflammatory cytokines and reducing oxidative stress. As a result, AuNPs have attracted considerable interest for their potential applications in treating inflammatory diseases and facilitating drug delivery, with promising implications for Alzheimer’s disease and other neuroinflammatory conditions. (Ref. 82).

#### Bariatric surgery

Recent studies provide compelling evidence that bariatric surgery can improve cognitive function among individuals with obesity. For instance, Alosco et al. demonstrated that patients exhibited sustained improvements in memory function 24 months post-surgery, suggesting that bariatric surgery may help prevent pathological cognitive decline and possibly the development of dementia (Ref. 97). Nozari et al. explored the impact of enhanced leptin signalling on cognitive function following bariatric surgery and proposed a potential mechanism for this cognitive improvement. Specifically, their findings suggest that cognitive benefits may be mediated by the restoration of the leptin JAK/STAT signalling pathway, driven by the reversal of inflammation-related processes in individuals with metabolic disorders (Ref. 81).

#### Exosome-based stem cell therapy

Exosomes derived from stem cells of various origins have shown the ability to cross the BBB, presenting a promising strategy for treating obesity-related cognitive impairment. The depletion of neural stem and progenitor cells (NSPCs) is thought to contribute to cognitive dysfunction in numerous age-related non-communicable diseases. A study by Natale et al. demonstrated that stimulating NSPCs with EV from NSPCs (exo-NSPC) restored IRS-1/Forkhead box protein O (FoxO) activation and mitigated the reduction in stem cell proliferation and senescence. Thus, intranasal administration of exogenous NSPCs could attenuate the damage caused by high-fat diet on hippocampal neurogenesis in adult mice by rebalancing between proliferating and senescent NSPCs (Ref. 83). Lu et al. showed that loading of EV with anti-TRIM21 oligonucleotides effectively inhibited TRIM21 in macrophages, thereby selectively alleviating HFD-induced inflammation and related metabolic disorders. This mechanism may be closely related to the role of the ubiquitin-binding enzyme E2M (UBE2M) in macrophage-driven obesity-related inflammation. HFD-induced obesity, IR and hepatic steatosis were significantly attenuated in mice lacking UBE2M in macrophages. This effect was achieved by reducing the pro-inflammatory activity of macrophages through decreased production of IL-1β. UBE2M deficiency inhibits the neddylation of TRIM21, which leads to a reduced degradation of the E3 ubiquitin ligase VHL. The degradation of HIF-1α by VHL, in turn, suppresses IL-1β production (Ref. 84). Zhong et al. reported that adipose-derived stem cells (ADSCs) in the inflammatory microenvironment produce glial cell-derived neurotrophic factor, which exerts neuroprotective effects in the nervous system. This is by promoting the PI3K/AKT signalling pathway to regulate M2 polarization in microglia (Ref. 85).

#### Recent advances in alternative therapeutic approaches

A recent investigation delving into the effects of probiotic mixtures on LTP found notable alterations in gut microbiota composition along with an increase in the expression of genes associated with brain inflammation and neuroplasticity (Ref. 86). Recent studies have suggested that strains of Bifidobacterium have a positive influence on cognitive processes (Ref. 87). Moreover, it has been shown that *A. muciniphila* can establish a symbiotic relationship with the host and exhibit biological functions such as anti-obesity, anti-inflammatory, anti-ageing and anti-neurodegenerative effects. However, since the study of functional diversity mediated by genomic and genotypic differences and metabolic differences among Akkermansia strains is still at a limited stage, further in-depth studies on the role of Akkermansia supplements in preventing and treating conditions such as inflammation, obesity, ageing and neurodegenerative diseases. Recent animal studies have shown that dimethylhexanedioate enhances cognitive performance in mice induced by HFD. Dietary restriction of leucine has been shown to improve obesity-related cognitive deficits by modulating the microbiota–gut–brain axis (Ref. 90). In the quinolinic acid-induced obesity model, butyric acid exerts its effects by ameliorating cognitive decline (Ref. 91). In an experimental mouse study, FSH was found to directly impact hippocampal and cortical neurons by accelerating the deposition of β-amyloid and Tau proteins, thereby impairing cognitive function in mice exhibiting AD characteristics. Inhibition of FSH activity, particularly through blocking the neuronal C/EBPβ-δ secretion pathway, successfully alleviated the AD-like phenotype in these mice. This reveals a causal relationship between elevated serum FSH levels and the pathophysiological process of menopausal AD, and provides an opportunity to treat AD and obesity using a single FSH inhibitor (Ref. 92). Another study validated that the deletion of the Ffar3 gene in Tg2576 mice prevented the development of cognitive deficits late in the disease. Remarkably, this improvement in cognitive impairment persisted despite severe metabolic challenges, such as exposure to HFD. The absence of FFAR3 also reinstates the heightened brain metabolism observed in Tg2576 mice, suggesting that FFAR3 may represent a potential novel therapeutic target for neurodegenerative diseases (Ref. 93).

## Conclusion

Recent epidemiological studies have shown that obesity is associated with cognitive impairment. Obesity is a risk factor for cognitive impairment. A large number of experimental studies have shown that obesity may lead to cognitive impairment by mechanisms involving IR, systemic inflammation and central inflammation, the immune system, microcirculatory changes, changes in neurotransmitters and synaptic plasticity. The adipose tissue–brain axis, the microbiota–gut–brain axis, and, in particular, the targeting effects on the brain of EV from AT and microbiota. Therefore, this paper also discusses a range of measures to improve obesity-related cognitive impairment, including optimization of lifestyle factors such as physical activity, diet and sleep, the use of anti-obesity drugs (GLP-1R agonists), anti-inflammatory drugs and consideration of bariatric surgery and extracellular vesicular stem cell therapy. Currently, our understanding of how specific components (e.g., proteins, lipids and nucleic acids) in the EV of adipose tissue and gut microbiota affect the brain in obese individuals remains poor. This is a knowledge gap in the research field. Therefore, the discovery of early EV biomarkers is important for the diagnosis and treatment of obesity-related cognitive impairment.

## Data Availability

The data presented in this study are available on request from the corresponding author.
